# Overview of state policies requiring smoking cessation therapy in psychiatric hospitals and drug abuse treatment centers

**DOI:** 10.1186/s12971-015-0059-2

**Published:** 2015-10-22

**Authors:** David Krauth, Dorie E. Apollonio

**Affiliations:** Department of Clinical Pharmacy, University of California, San Francisco, USA; Department of Clinical Pharmacy, Institute for Health Policy Studies, University of California, 3333 California Street, Suite 420, San Francisco, CA 94143 – 0613 USA

**Keywords:** Tobacco cessation, Alcohol, Substance abuse, Mental health, Health policy

## Abstract

**Background:**

Research demonstrates that individuals in substance abuse treatment are more likely to die from tobacco addiction than from their primary addiction, yet historically substance abuse treatment has not included treatment for tobacco addiction. The purpose of our study was to (1) review the diffusion of state policies mandating the provision of tobacco cessation treatment as a condition of state licensure in substance abuse treatment facilities and psychiatric treatment centers and (2) describe the current landscape of policies relating to tobacco cessation in state-licensed substance abuse treatment facilities and psychiatric treatment centers.

**Findings:**

We conducted a nationwide assessment of all 50 states from May 2013 - October 2014 to determine the progress each has made with developing a statewide tobacco cessation policy. We reviewed state government websites, conducted phone interviews with state regulatory agencies, and emailed state employees. Overall, 13 of 50 states (26 %) require tobacco cessation provision in alcohol, drug rehabilitation, and or mental health treatment centers, 6 states (12 %) are currently working towards a state policy, and 31 states (62 %) do not require tobacco cessation nor are working towards a state policy, though many of them have smoke free policies in both substance abuse centers and mental health wards.

**Conclusions:**

Our updated review of statewide smoking cessation policies in alcoholic, drug abuse, and mental health populations reveals that while clinical findings that affect population health may be well-publicized in the research community, these findings are not necessarily translated into policy. Further research on policy diffusion is needed.

## Findings

### Background

Patients undergoing treatment for mental health or substance abuse are disproportionately affected by smoking. Research demonstrates that individuals in substance abuse treatment are more likely to die from tobacco addiction than from their primary addiction, yet historically substance abuse treatment has not included treatment for tobacco addiction [1–3]. Among tobacco users receiving substance abuse treatment, the death rate from tobacco use was 1.5 times greater than from other addiction causes [1, 3].

Efforts to integrate simultaneous tobacco cessation therapy into chemical dependency and mental health treatment units have been hindered by several factors. Providers fear that quitting smoking simultaneously will compromise efforts to recover from other additions [4, 5], despite research showing that including tobacco cessation in addictions treatment does not compromise the treatment and sobriety of patients receiving simultaneous care for alcohol and drug abuse [6, 7]. Many healthcare providers also believe that the health risks from smoking are less important than the perceived benefits of smoking, which are thought to calm psychiatric patients and reduce the risk of relapse [Apollonio D, Philipps R, Bero L. 2012. "Interventions for tobacco cessation in people in treatment for or recovery from substance abuse." Cochrane Library v. 12, pp. 1–10]. Additional barriers include uncertainty regarding the best time to integrate smoking cessation treatment, and the fact that many individuals who staff drug abuse clinics and psychiatric wards are smokers themselves [Apollonio D, Philipps R, Bero L. 2012. "Interventions for tobacco cessation in people in treatment for or recovery from substance abuse." Cochrane Library v. 12, pp. 1–10].

Despite these challenges, some policymakers have started to recognize the need for concurrent treatment and have taken steps to shift the policy landscape in this area. In 2001, New Jersey implemented a policy that required substance abuse treatment centers to provide tobacco cessation treatment as a condition of licensure. In the first year, the state reported increases in tobacco abstinence among those in residential treatment, with no increase in irregular discharges. These findings were consistent with other literature suggesting concurrent treatment for tobacco and other substances is effective.

Recognizing this success, New York enacted its own tobacco cessation policy in 2008. In recent years, similar policies have been proposed in other states (Colorado, South Carolina, and Connecticut) but not enacted, and multiple institutions have chosen to offer smoking cessation therapy to clients on a voluntary basis. Nevertheless, there is no listing of current policies that address the issue, and no tracking of proposed policies.

The purpose of this study is two-fold: (1) to review the diffusion of state policies mandating the provision of tobacco cessation treatment as a condition of state licensure in substance abuse treatment facilities and psychiatric wards and (2) to describe the current landscape of policies relating to tobacco cessation in state-licensed substance abuse treatment facilities and psychiatric wards. We hypothesized that there may be increased reliance on research findings regarding tobacco cessation in the policymaking process, either directly or indirectly through the efforts of policy advocates and health care professionals, which would be demonstrated by the adoption of laws that provide greater protection against tobacco-related disease.

### Methods

We reviewed the public commentary and background information on proposed and existing policies to assess how the use of clinical evidence in the policymaking process is associated with legislative and regulatory outcomes. We searched state government websites and conducted phone interviews and/or email exchanges with the representatives of state regulatory agencies (1–2 contacts per state, depending on each agency’s regulatory authority) to determine the progress each state has made in developing policies of this nature. All data were collected between May 2013 and February 2014. We updated the database in October 2014 to account for any changes in a state’s status.

#### Study population

Our goal was to assess state policies that address tobacco addiction in marginalized populations, which suffer the greatest burden of tobacco-related disease. For the purposes of this study, we define these populations as individuals in treatment for substance use or mental health disorders. We focused on statewide policies because they represent large-scale systemic change that can immediately affect these groups. Localities or individual treatment centers may choose to require or offer smoking cessation therapy to clients on a case-by-case basis, but these efforts have limited scope. Federal policy does not currently address tobacco cessation in substance use or mental health treatment facilities. Although facilities that are federally accredited would be subject to any new rulemaking on this issue, many treatment centers that serve marginalized populations are not federally accredited, yet are subject to state policy. As a result, state policies are most likely to affect tobacco use in these groups.

#### Types of interventions

We defined smoking cessation therapy to include at least one of the following interventions.

- Counseling only, both individual and group sessions, delivered in a clinic setting for tobacco cessation purposes during the course of existing addictions treatment, or in addition to existing interventions for other addictions
- Nicotine replacement therapy (NRT) of all modalities (e.g. gum, patch), both prescription and over-the-counter, offered to individuals for tobacco cessation purposes during the course of existing addictions treatment
- Non-NRT pharmacology (e.g. varenicline or bupropion) offered to individuals for tobacco cessation purposes during the course of existing addictions treatment
- A combination of any of the above methods

#### Analysis

States were categorized as (a) requiring tobacco cessation provision in alcohol, drug rehabilitation and/or mental health treatment centers, (b) working towards a state policy, or (c) no state regulation or policy proposals.

### Results

As shown in Table 1, 13 of 50 states (26 %) require tobacco cessation provision in alcohol, drug rehabilitation, and or mental health treatment centers. Among the states mandating the provision of tobacco cessation services, five states require tobacco cessation only in substance use treatment centers, five states require tobacco cessation in both substance use treatment centers and mental health treatment centers, two states require cessation only in mental health treatment centers, and one state did not distinguish whether tobacco cessation is required in substance use treatment centers, mental health treatment centers, or both.

**Table 1:** Current regulations in states requiring tobacco cessation provision (*n* = 13/50, 26 %)

State	State policy
Alabama	The state psychiatric hospitals are tobacco free (effective 1/4/10) and smoking cessation therapies are provided. The Alabama Department Public Health offers smoking cessation education and whatever supports are necessary including medically supervised nicotine replacement, medications, support groups, and access to the Alabama quit-line. Additionally, the department certifies and contracts out with community providers who provide substance abuse services and requires that such entities shall directly or by referral provide a continuum of services for all clients/patients enrolled in each level of care that addresses tobacco use.
COMBINED	
Arkansas	In Arkansas the Department of Behavioral Health Services (DBHS) policy that all licensed substance abuse programs must be smoke-free under the 2009 Clean Indoor Act. In 2013, DBHS made a requirement that all contracted substance abuse centers provide tobacco cessation as a part of routine treatment in October 2013 and become tobacco-free on June 1, 2014.
SA	
Louisiana	In 2012, the Louisiana legislature passed house bill 80, now referred to Act 373, to prohibit smoking in psychiatric facilities of the Department of Health and Hospitals and to establish procedures for treatment of smokers with mental illness in such facilities.
MH	
Maryland	Requires as a condition of grant award that all patients are screened for nicotine dependence disorders and if identified must be included and addressed in the patient’s treatment plan. This requirement is for all American Society of Addiction Medicine (ASAM) levels of care.
COMBINED	
Massachusetts	Licensing requirements for the state’s substance abuse facilities include providing counseling and education. However, FDA-approved medications for tobacco cessation, an evidence-based standard of care since at least 2008, are not provided by the majority of substance abuse facilities. Also, if facilities receive special grant money in a given year, they sometimes provide nicotine patches to clients, but that is on a special basis and not part of standard licensing.
SA	
New Hampshire	All treatment contracts include the following statement: “the Contractor shall have policies and procedures for both client and Contractor staff, that not only creates a tobacco-free environment as required by law, but to offer tobacco cessation tools and programming.”
COMBINED	
New Jersey	“The contractee shall provide all services under this contract in a smoke-free environment. All treatment planning shall include education on tobacco use. The contractee shall work toward development of a tobacco-free program.”
UNKNOWN	
New York	As of 2008, all facilities treating drug or alcohol addiction must have programs in place to encourage clients to stop smoking. All treatment centers are required to be smoke-free, and staff members must abide by the ban.
SA	
North Carolina	The state’s psychiatric hospitals, treatment facilities and residential developmental centers are regulated by entities that require active treatment for nicotine addiction/tobacco treatment. All identified active problems require active treatment and the state is striving to refine and improve the way it provides active treatment in various domains.
MH	
Oklahoma	Statewide policy requires that the Oklahoma Department of Mental Health and Substance Abuse Services offer tobacco cessation for their clients.
COMBINED	
Oregon	Oregon has enacted regulations and policies that require tobacco cessation therapy services in state residential substance abuse treatment facilities and psychiatric hospitals. State administrative rules from the Oregon Health Authority's Addictions and Mental Health (AMH) Division require that both residential and outpatient facilities provide tobacco cessation services along with other substance abuse treatment and recovery services.
COMBINED	
Texas	Department of State Health Services (DSHS), who operates state psychiatric hospitals, has had a tobacco-free campus policy since 2004. Since 2010, providers who are contracted with DSHS to provide substance abuse treatment services have been required to provide treatment for tobacco dependency to clients who are being admitted for substance abuse treatment.
SA	
Vermont	The Vermont Department of Health Division of Alcohol and Drug Abuse Programs provides grants to substance abuse treatment centers throughout Vermont, which are referred to as “preferred providers.” In July 1, 2013, those grants included a provision requiring all preferred providers to adopt tobacco-free campus policies, including integrating tobacco addiction into treatment plans. Vermont does not have a state policy for mental health facilities.
SA	

Substance abuse (SA) only, mental health (MH) only, combined (substance abuse and mental health), unknown (New Jersey)

Table 2 shows that six states (12 %) are currently working towards a state policy. Among the states working towards a state policy, three states have proposed tobacco cessation be required only in substance use treatment centers, two states proposed tobacco cessation be required in both substance use treatment centers and mental health treatment centers, and one state proposed tobacco cessation be required only in mental health treatment centers.

**Table 2:** Current regulations in states working toward a state policy (*n* = 6/50, 12 %)

State	Proposed policy
Colorado	The Office of Behavioral Health (OBH) within the Colorado Department of Human Services currently does not have rules related to offering smoking cessation services. The Office, however, does collect data from all licensed substance use disorder (SUD) programs at time of admission and at discharge, and with annual updates for persons served in community mental health centers. The data systems used currently require the collection of tobacco use information for all people served.
SA	
	OBH does not contractually require the provision of smoking cessation services, though it has worked collaboratively with provider systems to identify model tobacco cessation policies and practices.
Iowa	In 2010–2012, the Iowa Department of Public Health (IDPH) Tobacco Cessation Division used some federal dollars to set up model tobacco cessation programs in three of the state's larger substance abuse residential units.
SA	
Maine	Maine has administered training opportunities consisting of 3 parts (helpline, educational, smoking cessation therapy) for behavioral health patients. The Department of Health and Human Services has also performed small scale and state level projects to evaluate smoking cessation in behavioral health settings, but no statewide law has been enacted that requires this service.
COMBINED	
Montana	There is no blanket Montana smoking cessation state policy. However, Montana has one state-managed hospital to treat mental illness and it instituted a tobacco free campus in 2009. This prohibits use and possession by everyone (patients, staff, and visitors) on their campus and provides for patient treatment. Montana also has one state-managed inpatient chemical dependency treatment facility. They also instituted a tobacco free policy in 2011. Additionally, there are two privately operated facilities with tobacco free policies: one is an inpatient facility, and the other is an outpatient facility.
MH	
New Mexico	New Mexico does not have a state policy for tobacco cessation services being offered at state residential substance abuse centers. However, the state’s tobacco control program staff are working with private and nonprofit providers to explore voluntary policies to provide tobacco cessation services as part of their package of services for their inpatient and outpatient clients.
SA	
Washington	Washington has worked for many years on the effort to integrate cessation and behavioral health with limited success. A few years ago, the Department of Social Health Services Division of Behavioral Health and Recovery (DBHR) implemented a contractual requirement that providers have smoke-free grounds as part of its efforts. Unfortunately, they were advised by the AG office that such a requirement would need an administrative or statutory mandate. While the current administrative code does not require smoke free grounds, it does require providers to screen clients for tobacco use, so the state has data on how many people entering chemical dependency treatment use tobacco.
COMBINED	

Substance abuse (SA) only, mental health (MH) only, combined (substance abuse and mental health)

The following 31 states (62 %) do not require tobacco cessation nor are working towards a state policy, though many of them have smoke free policies in both substance abuse centers and mental health wards: Connecticut, Ohio, Michigan, Wisconsin, Kentucky, Tennessee, Pennsylvania, South Carolina, Georgia, West Virginia, Mississippi, Nebraska, South Dakota, North Dakota, Arizona, California, Nevada, Alaska, Missouri, Wyoming, Kansas, Rhode Island, Florida, Idaho, Delaware, Virginia, Utah, Hawaii, Illinois, Indiana, and Minnesota. Table 3 lists steps these states have taken, without proposing or enacting statewide policies, to address smoking and smoking cessation in substance abuse centers and/or psychiatric wards.

**Table 3:** Current regulations in states not working toward a state policy (*n* = 31/50, 62 %)

State	Policy
Alaska	Tobacco Cessation is not a mandatory requirement for treatment plans for Inpatient Psychiatric Care, Residential Psychiatric Care, or SUD Residential Treatment. However, some of Alaska’s SUD programs have trained Tobacco Cessation specialists who include this component in the treatment program. However, there are state policies that promote the inclusion of tobacco cessation services and smoke/tobacco free campuses:
	1. Alaska Psychiatric Institute (Alaska’s state-run psychiatric hospital) is a smoke-free hospital. It has been smoke-free since 2007. Patients are provided smoking suppressants and smoking cessation services. Employees have access to tobacco cessation services through AlaskaCare (the state employee health insurance program).
	2. All behavioral health providers are required by state regulation to be accredited by Joint Commission, CARF, Council on Accreditation, or similar bodies. This results in a *de facto* requirement for tobacco use policies and tobacco cessation services. For example, the Joint Commission has quality measures related to screening for and treating tobacco use. CARF requires that organizations have policies related to use of tobacco products on campus and requiring screening for tobacco use. The Council on Accreditation also requires policies on tobacco use by employees, and requires organizations to provide information on health living choices (specifically including smoking cessation).
Arizona	Arizona has not yet ‘enacted’ any formal regulation with the exception that facilities have to abide by the clean indoor air act; no smoking is allowed within 20 ft of a building. The state has focused on concentrated work within behavioral health organizations to increase access to the state Quitline by the target populations.
California	The California Tobacco Control Program (CTCP) supports the treatment of tobacco dependences per the Treating Tobacco Use and Dependence: Clinical Practice Guidelines. The CTCP has also funded several educational trainings as part of its funded tobacco control projects working with the behavioral health (substance abuse and/or mental health) community and their partners to call attention for the need of this practice.
Delaware	Currently, the only tobacco cessation law in Delaware is designed to allow pregnant women and Medicaid beneficiaries to receive tobacco cessation counseling.
Florida	Florida has not enacted requirements or regulations for mental health, chemical dependency or other rehabilitation facilities or providers. However, many of these organizations have adopted no tobacco use policies supported by Florida’s clean indoor air laws. These laws have resulted in no tobacco use policies for in-patient psychiatric and chemical dependency services. They also restrict tobacco use in all indoor out-patient treatment facilities.
Georgia	The Georgia Tobacco Use Prevention Program has provided technical assistance towards the adoption of a model tobacco-free policy (adopted in 2010) within buildings and on the grounds of all of the Georgia mental health communities at the state and county level. Upon adoption of the model policy, discussions pertaining to developing cessation protocols for inpatient and outpatient consumers did not occur due to a lack of interest by the leadership of the mental health communities at the state and local levels. The goal of the Georgia Tobacco Use Prevention Program was to plan, implement and evaluate the model protocols for these populations/consumers in accordance with the U.S. Public Health Service Guidelines 2008 (updated version).
Idaho	Idaho currently does not have any statewide regulation or standards around cessation within substance abuse or psychiatric treatment facilities. The state owned mental health facilities do have smoke-free campus policies, however.
Kentucky	The need to integrate tobacco cessation services into substance abuse and mental health treatment services is becoming more widely recognized within Kentucky’s treatment provider community, and the Tobacco Program has sought to strengthen the connections between members of the Tobacco Prevention & Cessation Program and mental health and substance abuse treatment providers.
	Additionally, the Department for Behavioral Health, Developmental and Intellectual Disabilities has encouraged smoking cessation services be provided to clients with SUDs, and encouraged providers to go tobacco-free.
Michigan	Michigan’s state-owned psychiatric hospital campuses went smoke-free by law several years ago.
Missouri	There is no statewide policy but the policy for state owned facilities is that they be smoke free. Missouri is exploring tobacco cessation services with its Tobacco Treatment Handbook which the state has promoted at 3 different pilot sites.
Nevada	Although some Nevada-based providers do provide cessation, it is not mandated. What is mandated by the state’s Substance Abuse Prevention and Treatment Agency (SAPTA) with their providers is that there will be no smoking anywhere within the facility or on the external grounds of the facility. There can be no designated smoking areas either. That is by SAPTA policy. Nevada’s mental health agencies – Northern Nevada Adult Mental Health Services (NNAMHS), Southern Nevada Adult mental health Services (SNAMHS) and Lake’s Crossing Center have similar policies and requirements as well.
Pennsylvania	At this time no regulations or requirements for smoking cessation exist in Pennsylvania, but some of the residential facilities provide it voluntarily.
South Carolina	The South Carolina Department of Alcohol and Other Drug Abuse Services (DAODAS) has not enacted a tobacco cessation policy in its residential programs. However, the South Carolina Department of Health and Environmental Control has a Tobacco Quitline that clients can be referred to for services.
South Dakota	South Dakota does not have any formal policies requiring tobacco cessation treatment in substance abuse treatment facilities and psychiatric wards. However, the South Dakota Department of Health Tobacco Control Program just started a partnership with all the community mental health and substance abuse centers in South Dakota and plans to help them with setting buildings and grounds tobacco-free policies and educating their staff on promoting the use of the SD QuitLine.
West Virginia	The state provides educational information to its mental health facilities only.
Wisconsin	Wisconsin’s regulations of mental health and substance abuse provider agencies include a requirement to complete a comprehensive assessment of the clinical needs of their patients and to address identified needs with their clients based on an individualized treatment/recovery plan.
	Additionally, UW Madison’s Center for Tobacco Research and Intervention has been working with the Wisconsin Department of Health Services to provide training and technical assistance for the state’s mental health and substance abuse treatment providers to promote the best practice of integration of smoking cessation into treatment programs.
Wyoming	Wyoming has no statewide law, but 6 counties have gone smoke free.

Some states are omitted because current regulations do not address tobacco cessation in substance abuse centers/psychiatric wards in any capacity (Connecticut, Hawaii, Illinois, Indiana, Kansas, Minnesota, Mississippi, Nebraska, North Dakota, Ohio, Rhode Island, Tennessee, Utah, Virginia)

### Conclusion

Although we hypothesized that an increase in reliance on clinical research findings in the policymaking process could lead to the widespread adoption of state policy to protect against tobacco related disease, our exploratory analysis revealed this has not happened. Only one western state (Oregon) has enacted a statewide policy; suggesting that any policy diffusion has primarily occurred on the East Coast. Furthermore, most existing statewide regulations affect only substance use treatment centers and do not apply to mental health treatment centers.

In 2011, the National Survey of Substance Abuse Treatment Services (N-SSATS) published statewide data showing the percentage of substance abuse treatment facilities that provided tobacco cessation services, including tobacco cessation counseling, nicotine replacement therapy, and/or non-nicotine tobacco cessation medication [8]. The geographic differences presented in that survey are consistent with our finding that substance abuse centers offering tobacco cessation therapy were more likely to be located in the US Northeast than centers that did not offer these services. In New York, 83 % of substance abuse centers offered tobacco cessation services, giving it the highest percentage of any state to offer such services. Given that New York enacted a regulation requiring the provision of tobacco cessation as a condition of licensure for the state’s substance use treatment facilities in 2008, enforcement was not consistent as of 2011. Similarly, Texas enacted a state policy in 2010, yet only 44 % of its substance abuse centers were providing tobacco cessation in 2011. These examples raise concern about the extent to which statewide tobacco cessation policies that pass are enforced.

Overall, our updated description of statewide smoking cessation policies in alcohol, drug abuse, and mental health populations suggests that despite the well-publicized early success of the New Jersey policy, the overall diffusion of similar policies has been slow. A recent systematic review found that the continuing limited provision of tobacco cessation therapy in drug abuse and mental health treatment is not due to lack of knowledge about its positive effects [9]. States, however, have been slow to enact formal policies since New Jersey’s initiation in 2001, and New Jersey weakened its own policy several years later. The failure to enact or retain these policies may reflect limited resources, a lack of awareness of policies in other states, hostility from service providers who view tobacco cessation as a low priority, or a decision to “watch and wait” for the results of other states’ policies. In additional, even in states that do enact such policies, enforcement may be inconsistent across states and over time. Further study investigating what factors may prevent state policymakers from developing and implementing policies in this area is warranted.

### Study limitations

When reporting results we do not distinguish what form (counseling, nicotine replacement therapy, non-NRT pharmacology, etc.) of tobacco cessation therapy is required from each state, so are unable to compare which treatment is most common. We were unable to find this information via our search of state government websites and interviews with state regulatory agencies and employees.
